# Supplementary material for: From sequence to enzyme mechanism using multi-label machine learning
Source: BMC Bioinformatics. 2014 May 19;15:150. doi: 10.1186/1471-2105-15-150 (PMC4229970; doi:10.1186/1471-2105-15-150)
Supplement: Additional file 2 — Java code of ml2db. Additional file ml2db_code.tar.gz contains the Java source code to run the multi-label machine learning experiments and save the results to database. The code’s Javadoc is included. [file 1471-2105-15-150-S2.zip › additional file 2/ml2db/ml2db/doc/main/ml2db/al/learning/AlOutputManager.html]

AlOutputManager


JavaScript is disabled on your browser.


- Overview
- Package
- Class
- Use
- Tree
- Deprecated
- Index
- Help

- Prev Class
- Next Class

- Frames
- No Frames

- All Classes

- Summary:
- Nested |
- Field |
- Constr |
- Method

- Detail:
- Field |
- Constr |
- Method


main.ml2db.al.learning

## Class AlOutputManager

- java.lang.Object
- - uk.ac.standrews.utils.main.Initialised
  - - main.ml2db.mulan.learn.crosseval.CrossOutputManager
    - - main.ml2db.mulan.learn.traintest.TrainTestOutputManager
      - - main.ml2db.al.learning.AlOutputManager

- All Implemented Interfaces:
  :   uk.ac.standrews.utils.main.Initialisable

  ---

    

  ```
  public class AlOutputManager
  extends TrainTestOutputManager
  ```

  Class

  Version:
  :   14 Jul 2011

  Author:
  :   Luna De Ferrari ldf(at)st-andrews.ac.uk

- - ### Field Summary

    - ### Fields inherited from class uk.ac.standrews.utils.main.Initialised

      `m_initialisationIsCorrect`
  - ### Constructor Summary

    Constructors

    Constructor and Description

    `AlOutputManager(uk.ac.standrews.utils.main.database.DbManager resultsDbManager,
    java.lang.String resultsFolder,
    uk.ac.standrews.utils.main.database.Table table,
    AlExperimenter experimenter)`
  - ### Method Summary

    Methods

    | Modifier and Type | Method and Description |
    | --- | --- |
    | `AlExperimenter` | `getExperimenter()` |
    | `uk.ac.standrews.utils.main.database.TableRow` | `getRowValues(java.lang.Object[] results)` |
    | `AlEvaluationTable` | `getTable()` public TableRow getRowValues(Object[] results) { String run = (String) results[0]; int trainArffId = this.getExperimenter().getInputManager() .getTrainArffRecord().getArffId(); // for crossevaluation, same id for training and testing int testArffId = this.getTestId(); String startOfRun = (String) results[1]; String endOfRun = (String) results[2]; MulanLearner learnerName = this.getExperimenter().getLearner(); Evaluation evaluationMetrics = (Evaluation) results[3]; return EvaluationResultsRecord.createEvaluationRecord(run, startOfRun, endOfRun, learnerName, evaluationMetrics, Integer.toString(trainArffId), Integer.toString(testArffId), this.getTable().generateTableManager(this.getExperimentDbManager())); } |
    | `void` | `resetEvaluation()` |

    - ### Methods inherited from class main.ml2db.mulan.learn.traintest.TrainTestOutputManager

      `closeConnection, logEvaluation`
    - ### Methods inherited from class main.ml2db.mulan.learn.crosseval.CrossOutputManager

      `getEvaluationMeasures, getEvaluationResultsTable, getExperimentDbManager, getResultsDirectory`
    - ### Methods inherited from class uk.ac.standrews.utils.main.Initialised

      `initialisationIsCorrect, setInitialisation, setInitialisationIsCorrect, setInitialisationNotCorrect`
    - ### Methods inherited from class java.lang.Object

      `equals, getClass, hashCode, notify, notifyAll, toString, wait, wait, wait`

- - ### Constructor Detail


    - #### AlOutputManager

      ```
      public AlOutputManager(uk.ac.standrews.utils.main.database.DbManager resultsDbManager,
                     java.lang.String resultsFolder,
                     uk.ac.standrews.utils.main.database.Table table,
                     AlExperimenter experimenter)
      ```
  - ### Method Detail


    - #### getExperimenter

      ```
      public AlExperimenter getExperimenter()
      ```

      **Overrides:**
      :   `getExperimenter` in class `TrainTestOutputManager`


    - #### getRowValues

      ```
      public uk.ac.standrews.utils.main.database.TableRow getRowValues(java.lang.Object[] results)
      ```


    - #### getTable

      ```
      public AlEvaluationTable getTable()
      ```

      **Description copied from class: `CrossOutputManager`**

      public TableRow getRowValues(Object[] results) { String run = (String)
      results[0]; int trainArffId = this.getExperimenter().getInputManager()
      .getTrainArffRecord().getArffId();
      // for crossevaluation, same id for training and testing int testArffId =
      this.getTestId(); String startOfRun = (String) results[1]; String
      endOfRun = (String) results[2]; MulanLearner learnerName =
      this.getExperimenter().getLearner(); Evaluation evaluationMetrics =
      (Evaluation) results[3];
      return EvaluationResultsRecord.createEvaluationRecord(run, startOfRun,
      endOfRun, learnerName, evaluationMetrics, Integer.toString(trainArffId),
      Integer.toString(testArffId),
      this.getTable().generateTableManager(this.getExperimentDbManager())); }

      **Overrides:**
      :   `getTable` in class `CrossOutputManager`


    - #### resetEvaluation

      ```
      public void resetEvaluation()
      ```


- Overview
- Package
- Class
- Use
- Tree
- Deprecated
- Index
- Help

- Prev Class
- Next Class

- Frames
- No Frames

- All Classes

- Summary:
- Nested |
- Field |
- Constr |
- Method

- Detail:
- Field |
- Constr |
- Method
